# Monolithically integrated triaxial high-performance micro accelerometers with position-independent pure axial stressed piezoresistive beams

**DOI:** 10.1038/s41378-022-00474-z

**Published:** 2023-01-11

**Authors:** Mingzhi Yu, Libo Zhao, Shanshan Chen, Xiangguang Han, Chen Jia, Yong Xia, Xiaozhang Wang, Yonglu Wang, Ping Yang, Dejiang Lu, Zhuangde Jiang

**Affiliations:** 1 State Key Laboratory for Manufacturing Systems Engineering, International Joint Laboratory for Micro/Nano Manufacturing and Measurement Technologies, Xi’an Jiaotong University, 710049 Xi’an, China; 2 Xi’an Jiaotong University (Yantai) Research Institute for Intelligent Sensing Technology and System, Xi’an Jiaotong University, 710049 Xi’an, China; 3 Shandong Laboratory of Yantai Advanced Materials and Green Manufacturing, 265503 Yantai, China

**Keywords:** Sensors, Electronic devices

## Abstract

With the increasing demand for multidirectional vibration measurements, traditional triaxial accelerometers cannot achieve vibration measurements with high sensitivity, high natural frequency, and low cross-sensitivity simultaneously. Moreover, for piezoresistive accelerometers, achieving pure axial deformation of the piezoresistive beam can greatly improve performance, but it requires the piezoresistive beam to be located in a specific position, which inevitably makes the design more complex and limits the performance improvement. Here, a monolithically integrated triaxial high-performance accelerometer with pure axial stress piezoresistive beams was designed, fabricated, and tested. By controlling synchronous displacements at both piezoresistive beam ends, the pure axial stress states of the piezoresistive beams could be easily achieved with position independence without tedious calculations. The measurement unit for the *z-*axis acceleration was innovatively designed as an interlocking proof mass structure to ensure a full Wheatstone bridge for sensitivity improvement. The pure axial stress state of the piezoresistive beams and low cross-sensitivity of all three units were verified by the finite element method (FEM). The triaxial accelerometer was fabricated and tested. Results showing extremely high sensitivities (*x* axis: 2.43 mV/g/5 V; *y* axis: 2.44 mv/g/5 V; *z* axis: 2.41 mV/g/5 V (without amplification by signal conditioning circuit)) and high natural frequencies (*x/y* axes: 11.4 kHz; *z*-axis: 13.2 kHz) were obtained. The approach of this paper makes it simple to design and obtain high-performance piezoresistive accelerometers.

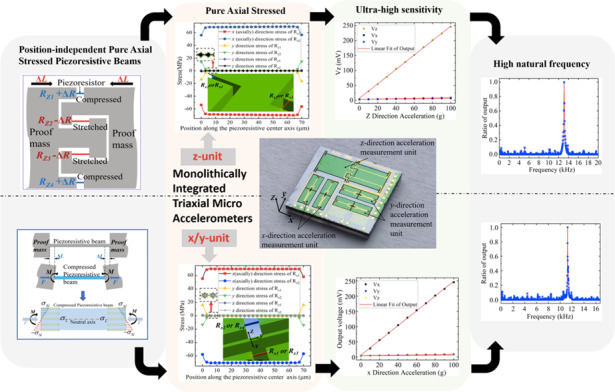

## Introduction

Piezoresistive accelerometers have been widely used in intelligent manufacturing, vehicle monitoring, and military fields^1–6^ due to their wide bandwidth, simple structures, and simple postprocessing circuits. The sensitivity and natural frequency are two critical parameters that determine the accelerometer’s performance in various applications. However, in a traditional cantilever beam structure or multibeam structure-based accelerometer^7–12^, the piezoresistor is directly arranged at the stress concentration region of the supporting beams, resulting in a direct coupling relationship between the natural frequency and the sensitivity, which makes it difficult to achieve high sensitivity and natural frequency at the same time.

Researchers have proposed structures of rigid supporting beams with relatively flexible piezoresistive beams to weaken the trade-off effect between the natural frequency and sensitivity^13^. Subsequent research has shown that pure axial stressed piezoresistive sensing beams can achieve high sensitivity without reducing the natural frequency^14–16^. However, to achieve pure axial stress, the piezoresistive sensing beams need to be specially located, which increases the difficulty in design. For example, our group recently designed a new type of piezoresistive accelerometer after complex design calculations to obtain a special position where the piezoresistive beam is in a pure axial stress state and then obtained a high sensitivity output^17,18^.

At present, most research has focused on single-axis accelerometers, which inevitably introduces installation errors and increases the size of the sensing system when measuring multidirectional vibrations by using several separately installed single-axis accelerometers. There are increasing applications in which multiaxial accelerations are needed for monitoring; therefore, developing a high-performance triaxial accelerometer is essential.

Extensive research has verified the advantages of multiaxial measuring capability in recent decades. Dong et al. designed a triaxial impact accelerometer^19^, in which the *x-* and *y* axes have a pure axial deformation piezoresistor, and the *z* axis is a dual-mass structure, which causes the natural frequency of the *z* axis to be half of the other axes. Hsieh et al. designed a stress isolation structure;^20^ the sensitivity of the tri-axis was 0.12–0.17 mV/g/V, and the natural frequency was only 1.57 kHz. Song et al. established a mathematical model of an eight-beam triaxial accelerometer^21^, in which the sensitivities of the three-axis were 0.209–1.247 mV/g/5 V, and the natural frequency was only ~2.7 kHz. Wang et al. developed a triaxial accelerometer with a double L-beam structure^22^, which consisted of four double L-beams. The triaxial sensitivities were 0.235–0.347 mV/g/5 V, and the natural frequencies were 8.9 kHz, 8.4 kHz, and 3.27 kHz. The above results revealed that the measurement consistency of the three sensitive axes is unsatisfactory, in which the low sensitivity and natural frequency limit its applications.

In this paper, a high-performance triaxial micro accelerometer is proposed. Benefitting from the position-independent pure axial stressed piezoresistive sensing beams, both high sensitivities and high natural frequencies in all axes are obtained. The realization of pure axial deformation does not require complex theoretical calculations. A novel interlocking proof mass structure is proposed in the *z* measurement unit to ensure a full Wheatstone bridge for sensitivity improvement. Then, numerical simulations based on the FEM are used to calculate the stress states and natural frequencies. Finally, the accelerometer is fabricated, and the performance is tested. The results indicate that the accelerometer has both high sensitivity, high natural frequency, and low cross-sensitivity. This strongly promotes the application of piezoresistive accelerometers in high-speed rail axle box fault diagnosis and precision machining condition monitoring.

## Materials and methods

### Model and structure

**Fig. 1:**
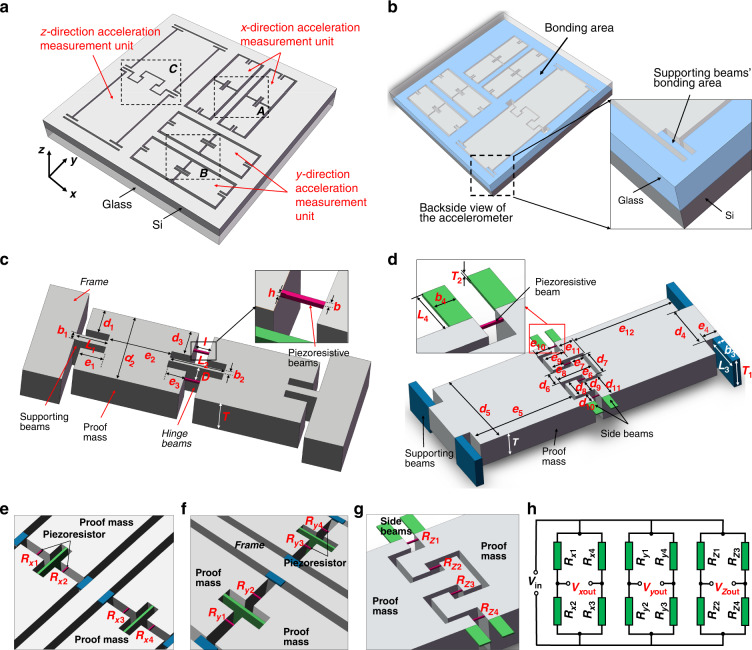
Structure of the monolithically integrated triaxial accelerometers. **a** Overview of the accelerometers. **b** Backside view of the accelerometer and the bonding area of the z measurement unit support beam. **c** 3D diagram of the subunits of the *x-* or *y* measurement unit. **d** 3D diagram of the z measurement unit. **e** Enlarged view of region A in (**a**), showing the piezoresistors position and numbering of the *x* measurement unit; **f** enlarged view of region B in (**a**); **g** enlarged view of region C in (**a**). **h** The output circuit

The accelerometer consists of *x*, *y*, and z measurement units that are used to measure the acceleration in the *x-*, *y-*, and z-directions, respectively, as shown in Fig. 1a and b. The *x* and *y* measurement units have the same structure, and they are perpendicularly placed. Each measurement unit includes two identical subunits.

As demonstrated in Fig. 1c, in each subunit, one end of the mass is fixed to the frame through the support beam, and another end is connected to the next mass end through the hinge beam and the piezoresistive beam. The piezoresistive beam is symmetrically distributed on both sides of the hinge beam. Figure 1d represents the *z* measurement unit, which consists of four supporting beams, two masses with specially designed interlocked proof mass ends, four piezoresistive beams, and four side beams. The bottom of the supporting beam is connected to the glass by anodic bonding (the magnified part of Fig. 1b). One end of the mass is connected to the support beam, and the other end is fixed to the frame through the side beams. Four piezoresistive beams are placed between the two masses. The two masses are specially designed with interlocked ends.

Figure 1e, f depicts the detailed layout of the piezoresistive beams. In Fig. 1e and f, there are two identical units with four piezoresistors on each unit for the *x*- and *y* measurement units, respectively. Figure 1g shows the layout of the piezoresistor for the *z* measurement unit. The special design of the interlocked structure is capable of opposite changes in *R*_*z*__1_ and *R*_*z*__4_ compared with *R*_*z*__2_ and *R*_*z*__3_. Therefore, a full Wheatstone bridge is achievable to improve the measurement sensitivity of *z*-direction acceleration. The output circuit, composed of the piezoresistors of each measurement unit in the nonworking state, is presented in Fig. 1h.

The values represented by the dimension numbers in Fig. 1c and d are shown in Table 1, which are also the dimension values when we simulate and fabricate the next stage.

**Table 1:** Dimensions of each part of the accelerometer

Parameter	Value/μm	Parameter	Value/μm	Parameter	Value/μm
*L*_*1*_	300	*d*_*6*_	560	*e*_8_	275
*L*_*2*_	500	*d*_*7*_	860	*e*_9_	345
*L*_*3*_	570	*d*_*8*_	425	*e*_10_	325
*L*_*4*_	320	*d*_*9*_	275	*e*_11_	325
*b*_*1*_	40	*d*_*10*_	195	*e*_12_	2620
*b*_*2*_	20	*d*_*11*_	520	*T*	405
*b*_*3*_	80	*e*_*1*_	230	*T*_*1*_	410
*b*_*4*_	75	*e*_2_	1155	*T*_*2*_	10
*d*_*1*_	410	*e*_3_	250	*b*	5
*d*_*2*_	1000	*e*_4_	150	*h*	10
*d*_*3*_	420	*e*_5_	2620	*l*	70
*d*_*4*_	1500	*e*_6_	670	*D*	110
*d*_*5*_	2000	*e*_7_	275		

### Working principle

According to Eq. (1), in the (100) crystal plane, the pure axial deformation of the piezoresistive beam, which undergoes zero-transverse stress (*σ*_*t*_ = 0), can maximize the use of deformation energy and greatly improve the sensitivity.

1 $$S = \frac{{\Delta R}}{{aR}}V_{{\mathrm{in}}} = \frac{{\pi _l\sigma _l - \pi _t\sigma _t}}{a}V_{{\mathrm{in}}}$$

where *S* is the sensitivity of the piezoresistive accelerometer, *R* is the resistance of the piezoresistor, Δ*R* is the change in resistance when the piezoresistor is subjected to stress, *a* is the acceleration applied to the accelerometer, *V*_in_ is the supply voltage to the accelerometer, *π*_*l*_ is the longitudinal piezoresistive coefficient, *π*_*t*_ is the transversal piezoresistive coefficient, *σ*_*l*_ is the axial stress on the piezoresistive beam, and *σ*_*t*_ is the transverse stress on the piezoresistive beam.

**Fig. 2:**
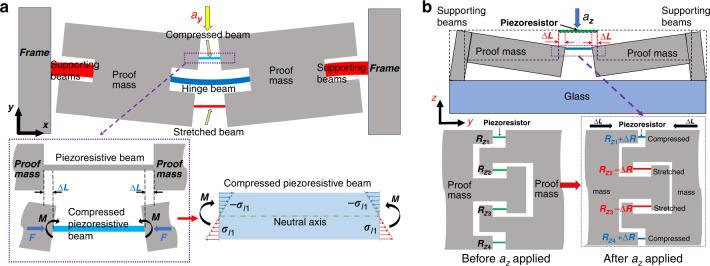
Each measurement unit working principle when subjected to acceleration. **a** Deformation of the corresponding measuring unit when subjected to acceleration in the *x-* or *y-*direction. **b** Deformation of the *z*-measuring unit when subjected to *z*-direction acceleration

Taking the *y* measurement unit as an example, as indicated in Fig. 2a. Two proof masses deflect synchronously, leading to deformation of the piezoresistive beams when acceleration in the *y-*direction is applied. Because the lateral displacements at both ends of the piezoresistive beam are identical, the piezoresistive beam has only axial displacement 2Δ*L* without lateral relative displacement.

Both axial force *F* and a pair of small moments *M* act on the piezoresistive beam. *M* makes the piezoresistive beam in a purely bending state (as shown in the lower right panel of Fig. 2a), there is no shear stress on the piezoresistive beam, and the tiny axial stresses *σ*_*l*1_ and −*σ*_*l*1_ caused by *M* show a gradient distribution along the transverse direction of the piezoresistive beam, bounded by the transverse midline, and the effects of *σ*_*l*1_ and −*σ*_*l*1_ on the piezoresistive output offset each other.

Therefore, the piezoresistors are only subjected to pure axial stress, and Eq. (1) can be expressed as:

2 $$S = \frac{{\pi _l\sigma _l}}{a}V_{{\mathrm{in}}}$$

Whenever the position of the piezoresistive beam changes, the piezoresistive beam always satisfies the state of pure axial deformation, and there is no need to go through complicated calculations, as in previous studies^13–18^. The piezoresistive beam exhibits double-end tensile deformation, and the axial stress is larger than that in previous studies, which consequently ensures a higher sensitivity.

Figure 2b illustrates the deformation of the *z* measurement unit under acceleration *a*_z_ and the deformation of the corresponding piezoresistors. The two proof masses deflect in the *z*-direction caused by inertia, and then the relative displacement in the *y-*direction (axial direction of the piezoresistor) is generated. Similar to the *x*/*y* measurement unit, there is no transverse displacement due to the synchronous deflection, which results in the pure axial deformation of the piezoresistive beams. Due to the specially designed interlocking proof mass end, *R*_*z*1_ and *R*_*z*4_ are subject to compressive stress, and *R*_*z*2_ and *R*_*z*3_ are subject to tensile stress. Therefore, the four piezoresistors can form the Wheatstone full bridge for output.

## Results and discussion

### FEM results

The accelerometer performance is analyzed by the finite element software Ansys Workbench. The material parameters of the Si used are density *ρ* = 2330 kg/m^3^, elastic modulus E = 1.7E11 Pa, and Poisson’s ratio *v* = 0.28. The boundary conditions are as follows: fixed constraints are applied to the bottom surface of the chip glass, and the applied load is 100 g acceleration. Then, stress analysis and modal analysis of each measurement cell of the accelerometer are performed using the default mechanical grid of the software.

**Fig. 3:**
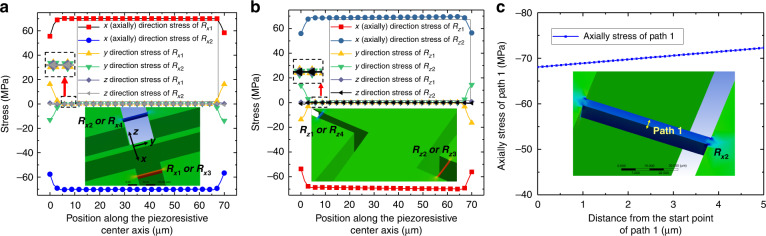
Stress distribution of the piezoresistive beams. **a** Stress distribution of the *x* or *y* measurement unit when *a*_*x*_ = 100 g or *a*_y_ = 100 g. **b** Stress distribution of the *z* measurement unit when *a*_z_ = 100 g. **c** The axially stress distribution gradients along the Path 1

Figure 3 reveals the pure axial stress state of the piezoresistive beam of each measurement unit of the triaxial accelerometer. The *x*- and *y* measurement units have the same stress state when 100 g sensitive direction acceleration is applied, taking the *x* measurement unit as an example. The stress distribution is uniform, as shown in Fig. 3a. The inset shows the stress distribution. The stresses are mainly concentrated in the piezoresistive beam, and the stresses in the proof mass, support beam and hinge beam are close to zero. The axial stress (i.e., *x-*direction) in the piezoresistive beam is ~70.1 MPa, and neglecting the stress inhomogeneity at the connection between the piezoresistive beam and the mass, the transverse stress (i.e., *y-*direction) is close to zero (<0.003 MPa), which indicates that the piezoresistive beam is in the pure axial stress state, which maximizes the use of deformation energy and greatly improves the sensitivity.

Figure 3b depicts the stress state of the *z* measurement unit when it is subjected to acceleration in the *z*-direction. The pure axial stress is 69.3 MPa, and the transverse stress is close to zero. Figure 3c shows the axial stress gradient along the width direction of the piezoresistive beam in the *x* and *y* measurement units mentioned in Fig. 2a (Path 1 in the illustration). This stress gradient has no effect on the sensor output, as described earlier in Fig. 2a.

**Fig. 4:**
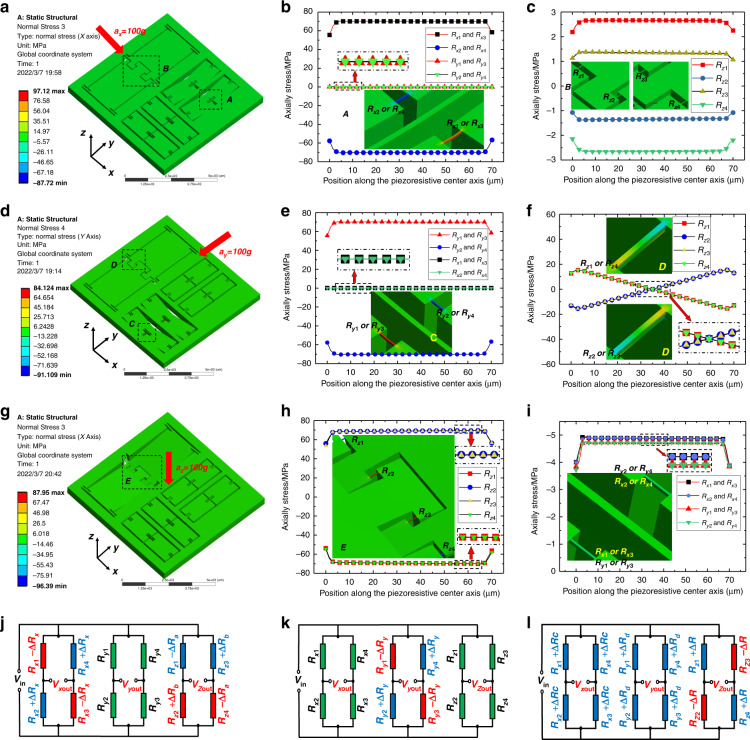
Sensitivity and cross-sensitivity analysis when 100 g acceleration in each direction is applied. **a** The deformation when *x*-direction acceleration *a*_*x*_ = 100 g, and stress distribution of (**b**) *x*/*y* and (**c**) *z* measurement unit; **d** the deformation when *a*_*y*_ = 100 g, and stress distribution of (**e**) x/y and (**f**) *z*-measuring unit; **g** the deformation when *a*_*z*_ = 100 g, and stress distribution of (**h**) *z* and (**i**) x/y-measuring unit. **j** The output circuit under *x-*direction acceleration. **k** The output circuit under y-direction acceleration. **l** The output circuit under *z-*direction acceleration

Figure 4 illustrates the stress distribution of the piezoresistive beams in each measurement unit when the accelerometer is subjected to acceleration in three directions. Figure 4j–l denotes the output circuits under the corresponding acceleration.

Figure 4a shows the stress distribution under the acceleration of _*ax*_ = 100 g. The enlarged images of regions A and B are the insets in Fig. 4b and c. The stress is mainly concentrated on the piezoresistive beams of the *x* measurement unit. It can be observed in Fig. 4b that the stresses on *R*_*x*1_ and *R*_*x*3_ are tensile stresses with values of 70.1 MPa, while the stresses of *R*_*x*2_ and *R*_*x*4_ are compressive stresses with a value of −70.1 MPa. Assuming the resistance change of *R*_*x*1_ and *R*_*x*3_ caused by tensile stress is Δ*R*_*x*_, the resistance changes of *R*_*x*2_ and *R*_*x*4_ caused by compressive stress should be −Δ*R*_*x*_. The stresses of the four piezoresistive beams in the *y* measurement unit are approximately zero (<0.018 MPa), which indicates that the resistance change is approximately zero. As shown in Fig. 4c, the stresses of the piezoresistive beams in the *z* measurement unit are 2.66 MPa (*R*_*z*1_), −1.34 MPa (*R*_*z*2_), 1.34 MPa (*R*_*z*3_), and −2.66 MPa (*R*_*z*4_), respectively. For simplicity, the resulting resistance changes of *R*_*z*1_~*R*_*z*4_ are denoted as −Δ*R*_*a*_, Δ*R*_*b*_, −Δ*R*_*b*_, and Δ*R*_*a*_. The resistance of unstressed beams is denoted as *R*. In Fig. 4j, the output voltages of the triaxial accelerometer are calculated:

3 $$\left\{ \begin{array}{l}V_{{\mathrm{xout}}} = \frac{{\Delta R_x}}{R}V_{{\mathrm{in}}}\\ V_{{\mathrm{yout}}} = 0\\ V_{{\mathrm{zout}}} = \frac{{V_{{\mathrm{in}}}}}{4}(\frac{{R_{z1}\, -\, \Delta R_a}}{{R_{z1}}} - \frac{{R_{z2}\, + \,\Delta R_b}}{{R_{z2}}} - \frac{{R_{z3}\, - \,\Delta R_b}}{{R_{z3}}} + \frac{{R_{z4} \,+\, \Delta R_a}}{{R_{z4}}})\\ {{{\mathrm{ = }}}}\frac{{V_{{\mathrm{in}}}}}{4}(\frac{{\Delta R_a - \Delta R_a + \Delta R_b - \Delta R_b}}{R}) = 0\end{array} \right.$$

According to Eq. (2), the simulation sensitivity is:

4 $$\left\{ \begin{array}{l}S_{{\mathrm{xFEM}}} = \frac{{V_{{\mathrm{xout}}}}}{{a_x}} = \frac{{\pi _l\sigma _l}}{{a_x}}V_{{\mathrm{in}}} = \frac{{\pi _{44}\sigma _{lx}}}{{2a_x}}V_{{\mathrm{in}}} = 2.42\;{\mathrm{mV}}/{\mathrm{g}}/{\mathrm{5V}}\\ S_{{\mathrm{yFEM}}} = 0\\ S_{{\mathrm{zFEM}}} = 0\end{array} \right.$$

where $\pi _{44} = 1.38 \times 10^{ - 9}{\mathrm{m}}^2/{\mathrm{N}}$ is the P-type doping piezoresistive coefficient;^23,24^ *V*_in_ = 5 V is the supply voltage; *σ*_*lx*_ = 70.1 MPa is the axial stress of the *x* measurement unit when *a*_*x*_ = 100 g.

Figure 4d shows the stress distribution of the triaxial accelerometer under the acceleration of *a*_*y*_ = 100 g. The enlarged images of regions C and D are the illustrations in Fig. 4e, f. The stress state of the *y* measurement unit is the same as the previous stress state of the *x* measurement unit. The stress of the four piezoresistive beams of the *x* measurement unit is zero. As shown in Fig. 4f, in the *z-*measuring unit, the stress states of the front and rear halves of the piezoresistive beam along the axial direction are opposite; therefore, the piezoresistor value is unchanged. In Fig. 4k, under the acceleration of *a*_*y*_, the output of the accelerometer is:

5 $$\left\{ \begin{array}{l}V_{{\mathrm{xout}}} = 0\\ V_{{\mathrm{yout}}} = \frac{{\Delta R_y}}{R}V_{{\mathrm{in}}}\\ V_{{\mathrm{zout}}} = 0\end{array} \right.$$

From Eq. (2), the simulation sensitivity under *a*_*y*_ is:

6 $$\left\{ \begin{array}{l}S_{{\mathrm{xFEM}}} = 0\\ S_{{\mathrm{yFEM}}} = \frac{{V_{{\mathrm{yout}}}}}{{a_y}} = \frac{{\pi _l\sigma _l}}{{a_y}}V_{{\mathrm{in}}} = \frac{{\pi _{44}\sigma _{ly}}}{{2a_y}}V_{{\mathrm{in}}} = 2.42\;{\mathrm{mV}}/{\mathrm{g}}/{\mathrm{5V}}\\ S_{{\mathrm{zFEM}}} = 0\end{array} \right.$$

where *σ*_*ly*_ = 70.1 MPa is the axial stress of the *y* measurement unit when applying acceleration *a*_*y*_ = 100 g.

Figure 4g shows the stress distribution under the acceleration of *a*_*z*_ = 100 g. The enlarged images of region E in Fig. 4g are the illustrations in Fig. 4h. The stress is mainly concentrated on the piezoresistive beams of the *z* measurement unit. It can be observed in Fig. 4h that the stresses of the *z-*measuring unit piezoresistive beams *R*_*z*1_ and *R*_*z*3_ are identical with a value of −69.3 MPa, while *R*_*z*2_ and *R*_*z*4_ are 69.3 MPa. The resistance changes of *R*_*z*1_ and *R*_*z*3_ caused by the stress are denoted as Δ*R*_*z*_, and the resistance changes of *R*_*z*2_ and *R*_*z*4_ are −Δ*R*_*z*_. In Fig. 4i, the stresses of the piezoresistive beams in both the *x* and *y* measurement units are compressive stresses, and the Wheatstone bridge has no output at this time.

In Fig. 4l, under the acceleration of *a*_*z*_, the output of each measurement unit of the accelerometer is:

7 $$\left\{ \begin{array}{l}V_{{\mathrm{xout}}} = 0\\ V_{{\mathrm{yout}}} = 0\\ V_{{\mathrm{zout}}} = \frac{{\Delta R_z}}{R}V_{{\mathrm{in}}}\end{array} \right.$$

From Eq. (2), the simulation sensitivity under *a*_*z*_ is:

8 $$\left\{ \begin{array}{l}S_{{\mathrm{xFEM}}} = 0\\ S_{{\mathrm{yFEM}}} = 0\\ S_{{\mathrm{zFEM}}} = \frac{{V_{{\mathrm{zout}}}}}{{a_z}} = \frac{{\pi _l\sigma _l}}{{a_z}}V_{{\mathrm{in}}} = \frac{{\pi _{44}\sigma _{lz}}}{{2a_z}}V_{{\mathrm{in}}} = 2.39\;{\mathrm{mV}}/{\mathrm{g}}/{\mathrm{5V}}\end{array} \right.$$

where *σ*_*lz*_ = 69.3 MPa is the axial stress of the *z* measurement unit when applying acceleration *a*_*z*_ = 100 g.

The above discussion confirms that the designed triaxial accelerometer theoretically has no cross-signal output, but due to a manufacturing error, residual stress, and material defects, the actual chip will have slight cross-sensitivity.

**Fig. 5:**
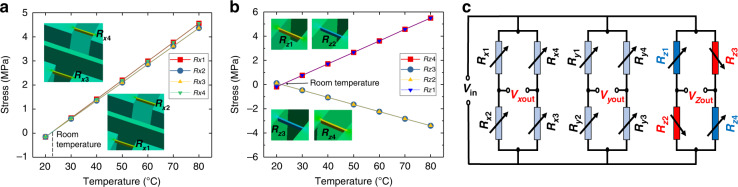
Effect of temperature on accelerometer output. Variation of the average thermal stress of the **a** *x* measurement unit and **b** z measurement unit piezoresistive beam with temperature. **c** Variation of piezoresistors of each measuring unit with temperature

Figure 5 presents the effect of temperature on the output of the accelerometer. Figure 5a shows that the average stress of the *x*-unit piezoresistive beam changes with temperature as *R*_*x*__1_ = *R*_*x*__4_ and *R*_*x*__2_ = *R*_*x*__3_. In Fig. 5c, there is no voltage output from the *x*-unit Wheatstone bridge at this time, so theoretically, the temperature will not influence the *x*-unit output. This conclusion is also applicable to the *y*-unit. Figure 5b shows that with the change in temperature, the average stress of the *z*-unit piezoresistive beam changes as *R*_*z*__1_ = *R*_*z*__4_ and *R*_*z*__2_ = *R*_*z*__3_. In Fig. 5c, the *z*-unit Wheatstone bridge configuration is different from the *x* and *y* units, so the temperature will affect the output of the *z-*unit at this time. In addition, the sensor package also affects the accelerometer output, which will be analyzed later.

**Fig. 6:**
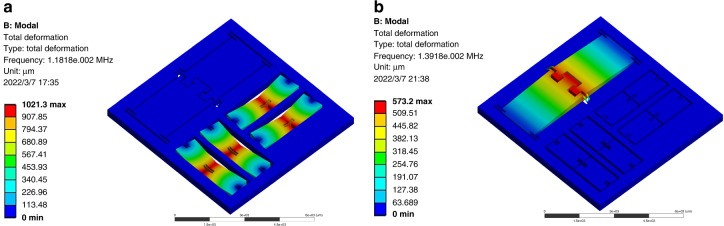
Dynamic performance of the accelerometer. **a** The first vibration mode. **b** The second vibration mode

Here, modal analysis is used to study the natural frequency and vibration modes of the structure. As shown in Fig. 6a, the first-order model is the vibration of the *x* and *y* measurement units. The vibration mode is in-plane vibrations along the sensitive directions with a frequency of 11.8 kHz. As illustrated in Fig. 6b, the second-order mode is the mode of the *z*-measuring unit, whose vibration mode is out-of-plane vibration along the *z*-direction with a frequency of 13.9 kHz.

### Fabrication and packaging

**Fig. 7:**
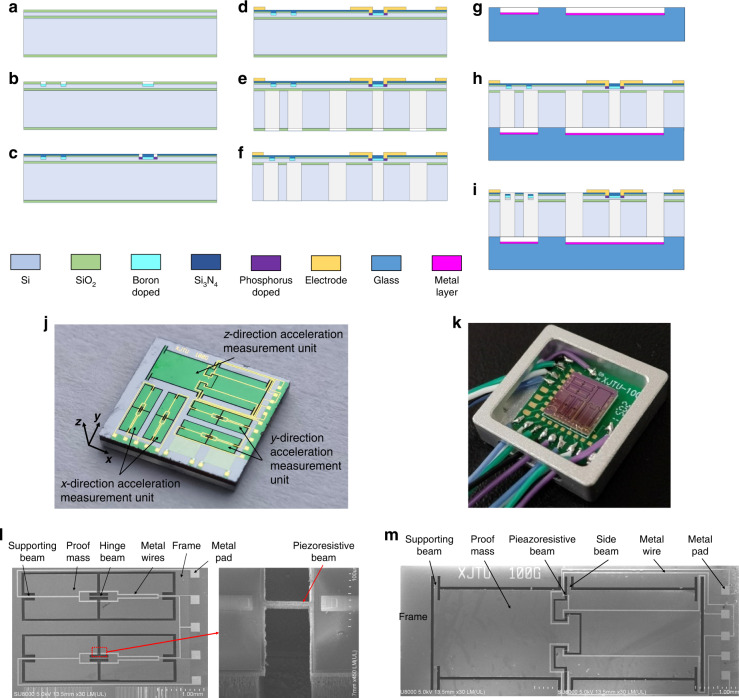
Fabrication processes and the accelerometer chip and package. **a** Thermal oxidization. **b** Light boron diffused. **c** Heavy boron diffusion. **d** Metal wire and pad. **e** DRIE for backside. **f** Remove buried oxygen layer. **g** Glass corrosion and Sputtering metal. **h** Anodic bonding. **i** ICP etching in front side. **j** Designed accelerometer. **k** The package. **l** SEM image of *x* or *y* measurement unit. **m** SEM image of z measurement unit

The fabrication process is shown in Fig. 7a–i. Accelerometer chip fabricated by a silicon-on-insulator (SOI) wafer. The parameters of the SOI silicon wafer are as follows: N-type (100) crystal orientation, the resistivity of 1–10 Ω cm, the device layer thickness of 10 μm, buried oxygen layer thickness of 1 μm, and substrate layer thickness of 400 μm.

- The SOI wafer is thermally oxidized on both sides to form a SiO_2_ layer with a thickness of 300 nm;
- The SiO_2_ layer is patterned, and then lightly doped with boron ions. The implantation dose is 1 × 10^16^ cm^−2^, and the resistance of this area is ~300 Ω/□;
- The SiO_2_ layer is removed and 400 nm SiO_2_/SiN_x_ is deposited and patterned. Then, the wafer is doped with boron ions with an implantation dose of 5 × 10^14^ cm^−2^, which results in a resistance of ~10 Ω/□, to form the ohmic contact.
- The mask layer of the ohmic contact region is removed, and a Ti/Al (200 Å/3000 Å) layer is deposited by PVD technology to form the metal leads;
- The SiO_2_ on the backside is patterned, and the silicon is etched using DRIE technology with a depth of 400 μm to form the backside structure of the accelerometer.
- The RIE process is used to remove the buried oxide layer from the backside.
- Etching BF33 and sputtering 100 nm gold is performed to prevent electrostatic adsorption during anodic bonding;
- The anodic bonding process is carried out; the temperature is 400 °C, the voltage is 1000 V, and the time is 1 h.
- Si is etched by ICP technology with an etching depth of 10 μm to form the front-side structure.

The fabricated accelerometer is pictured in Fig. 7j. The chip size is 7.2 mm × 7.4 mm × 0.91 mm (length × width × thickness). The measured resistance value of the piezoresistor is ~4.5 kΩ. The accelerometer chip is packaged on a PCB and housed with an aluminum alloy, as shown in Fig. 7k. The SEM images of the *x*, *y,* and *z* measurement units are displayed in Fig. 7l and m.

### Experimental test results

The static performance and dynamic performance of the designed triaxial accelerometer are tested, including sensitivity, cross-sensitivity, zero-g time drift, temperature coefficient offset (TCO), and natural frequency.

**Fig. 8:**
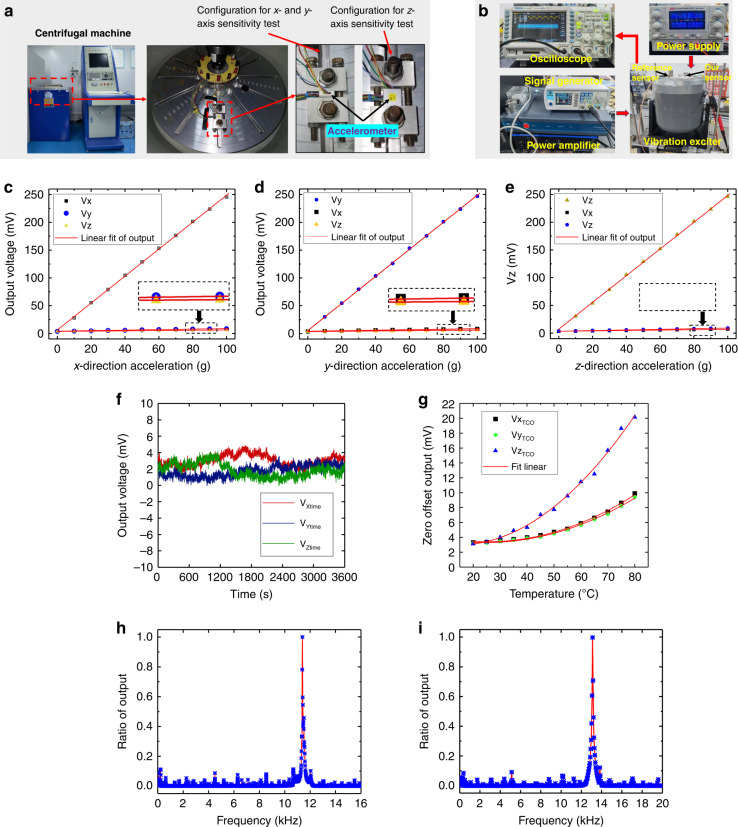
Experimental platform, Static and dynamic performance of the accelerometer. **a** Centrifugal test system for static performance testing. **b** The dynamic calibration system. **c** Output voltage in the *x*-direction acceleration. **d** Output voltage in the *y*-direction acceleration. **e** Output voltage in the *z*-direction acceleration. **f** The zero-g offset characteristic of the accelerometer. **g** Temperature coefficient of zero-g offset (TCO). **h** The characteristic curves of the resonance frequency of *x-* or *y* measuring units. **i** The characteristic curves of the resonance frequency of *z*-measuring unit

Sensitivity tests are carried out by using the centrifugal method. The test platform is presented in Fig. 8a. The output signals of different sensitive axes are tested by changing the installation direction, and the acceleration is controlled by controlling the rotation speed of the centrifuge and the installation position of the accelerometer. During the test, the supply voltage is 5 V. The output voltage signal of the accelerometer is depicted in Fig. 8c–e. The voltage output signal is not amplified by the signal processing circuit.

As shown in Fig. 8c, under *x*-direction acceleration, the sensitivity S_*x*_ of the *x* measurement unit is 2.43 mV/g/5 V, and the maximum nonlinear error is 1.42% FS. The cross-axis sensitivity S_*xy*_ of the *y* measurement unit is 0.037 mV/g/5 V, which is 1.52% of S_*x*_; the cross-axis sensitivity S_*xz*_ of the *z* measurement unit is 0.015 mV/g/5 V, which is 0.62% of S_*x*_.

As demonstrated in Fig. 8d, under *y*-direction acceleration, the sensitivity S_*y*_ of the *y* measurement unit is 2.44 mV/g/5 V, and the maximum nonlinear error is 1.21% FS. The cross-axis sensitivity S_*yx*_ of the *x* measurement unit is 0.038 mV/g/5 V, which is 1.56% of S_*y*_; the cross-axis sensitivity S_*yz*_ of the *z* measurement unit is 0.012 mV/g/5 V, which is 0.49% of S_*y*_.

As illustrated in Fig. 8e, under the *z*-direction acceleration, the sensitivity S_*z*_ of the *z* measurement unit is 2.41 mV/g/5 V, and the maximum nonlinear error is 1.83% FS. The cross-axis sensitivity S_*zx*_ of the *x* measurement unit is 0.016 mV/g/5 V, which is 0.66% of S_*z*_; the cross-axis sensitivity S_*zy*_ of the *z* measurement unit is 0.028 mV/g/5 V, which is 1.16% of S_*z*_.

The zero-g time drift characteristics are measured at a temperature of 23 °C in the incubator. The sampling interval is 1 s, and the sampling time is 2 h. The output signal curve of each measurement unit is shown in Fig. 8f. The zero-time drift of each measurement unit of the accelerometer is less than 0.82% FS/h.

The temperature coefficient offset (TCO) is measured in the incubator with a heating interval of 10 °C, and a holding time of 60 min is adopted for each interval. The TCO value distribution is shown in Fig. 8g. According to the definition:

9 $$TCO = \frac{{V_0(T_2) - V_0(T_1)}}{{V_{FS}(T_1)(T_2 - T_1)}}$$

where *V*_0_*(T*_2_*)* and *V*_0_*(T*_1_*)* are the sensor zero-point offset voltages under the temperature of *T*, respectively. *V*_FS_*(T*_1_*)* is the full-scale output voltage under the temperature of *T*_1_. The calculated TCO of the *x-*, *y-*, and *z* measurement units is 0.00066 FS/°C, 0.00064 FS/°C, and 0.00118 FS/°C, respectively.

The TCO of the *z* measurement unit is larger than that of the *x* and *y* measurement units, which is caused by the inconsistent stresses in the *z* measurement unit piezoresistive beam due to temperature changes. The TCO of the *z* measurement unit in this paper is compared with literature and products in Table 3. The *z*-axis TCO is comparable to that of the available literature and products and therefore does not affect subsequent applications.

However, the thermal stress growth trend of the *x*, *y*, and *z* measurement cells in Fig. 8g is not consistent with the results in Fig. 5. The main reason is that the temperature change makes the sensor package generate thermal stresses that act on the sensor structure. For example, the thermal expansion of the adhesive, PCB, and metal shell. These different materials make the TCO grow nonlinearly in the actual test. This makes it very difficult to analyze the effect of temperature on the mechanical behavior of accelerometers, and we will conduct further research in this area.

The dynamic output characteristics are measured by the sweep frequency method. As shown in Fig. 8b, the supply voltage is 5 V, and the output signal is collected by the oscilloscope. The vibration of the shaking table is controlled by a signal generator and a power amplifier. The accelerometer is continuously excited by a small acceleration from low frequency to high frequency so that the accelerometer slowly enters the resonant state. Finally, the natural frequency is obtained according to the frequency sweep characteristics. The natural frequencies of the *x* and *y* measurement units are similar, with a value of 11.4 kHz, as indicated in Fig. 8h. The natural frequency of the *z* measurement unit is 13.2 kHz, as presented in Fig. 8i.

The performance parameters are summarized in Table 2. The sensitivity and natural frequency measured by the experiment are consistent with the simulation.

**Table 2:** The characteristic parameters of the triaxial accelerometer

Parameters	*x* measurement unit		*y* measurement unit		*z* measurement unit		Unit
	FEM results	Test results	FEM results	Test results	FEM results	Test results	
Range	±100	±100	±100	±100	±100	±100	g
Sensitivity	2.42	2.43	2.42	2.44	2.39	2.41	mV/g/5 V
Natural frequency	11.8	11.4	11.8	11.4	13.9	13.2	kHz
Nonlinearity		1.42		1.21		1.83	%FS
Cross-sensitivity	0	S_xy_ = 0.037	0	S_yx_ = 0.038	0	S_zx_ = 0.016	mV/g/5 V
	0	S_xz_ = 0.015	0	S_yz_ = 0.012	0	S_zy_ = 0.028	
Zero-g offset		<0.82		<0.82		<0.82	%FS/h
TCO		0.00066		0.00064		0.00118	/°C

To facilitate comparison with other accelerometers, the FOM, as a figure of merit, is introduced to measure the benchmark performance of accelerometers:^25^

10 $${\mathrm{FOM}} = Sf^2$$

where *S* is the sensitivity of each measurement unit and *f* is the first-order natural frequency of each measurement unit. It can be seen in Table 3 that our structure achieves significantly higher sensitivity than other works while maintaining a high natural frequency, which demonstrates that our idea of achieving a piezoresistive pure axial stress state without complex calculations effectively improves the performance of accelerometers. The FOM of the proposed triaxial accelerometer is much higher than the accelerometers in the published works.

**Table 3:** Performance parameter comparison with the published literature

Source	Range (g)	Chip size (mm)	Sensitivity (mV/g/5 V)			Natural frequency (kHz)			Max cross-sensitivity (%)	Max TCO (°C^−1^)	FOM		
			*x*	*y*	z	*x*	*y*	z			*x*	*y*	z
Our work	±100	7.2 × 7.4 × 0.9	2.43	2.44	2.41	11.4	11.4	13.2	1.56	0.00118	6.34e4	6.31e4	8.4e4
Wang et al.^9^	±100	5 × 5 × 0.8			0.66			14.8	1.04				2.89e4
Liu et al.^24^	±100	5 × 5 × 0.82			0.908			13.31	6.88	0.00229			3.22e4
Sankar et al.^26^	±13	8 × 8 × 1.27			0.675			2.54	0.62	0.00031			8.71e2
Song et al.^21^	±10	6.6 × 6.6 × 0.9	0.209	0.212	1.247	3.97	3.98	2.7	4.31		6.58e2	6.72e2	1.82e3
Wang et al.^22^	±15	4 × 4 × 1	0.302	0.235	0.347	8.9	8.4	3.27	8.09		4.78e3	3.32e3	7.42e2
Endevco7264^a^	±200				1.25			6	3	0.00095			9e3
Endevco7268^b^	±500		0.4	0.4	0.4	17	17	17	3	0.00120	2.3e4	2.3e4	2.3e4

^a^Endevco® Inc, https://buy.endevco.com/nx/SearchResults?q = 7264, 2022.11.06.

^b^Endevco® Inc, https://buy.endevco.com/Products?m = 7268c-500-360, 2022.11.06.

## Conclusion

In summary, a novel monolithically integrated triaxial accelerometer with position-independent pure axial stressed piezoresistive beams is proposed for extremely high sensitivity, high natural frequency and low cross-sensitivity. By controlling synchronous displacements at both piezoresistive beam ends, the pure axial stress state is achieved and does not depend on the position of the piezoresistive beam. This method greatly simplifies the design process. The *z* measurement unit is creatively designed as an interlocking structure to ensure a full Wheatstone bridge. The pure axial stress states, high sensitivity, and high natural frequency are verified by an FEM simulation and tests. The accelerometer shows that the sensitivities of the *x*, *y*, and *z* measurement units are 2.43 mV/g/5 V, 2.44 mV/g/5 V, and 2.41 mV/g/5 V, respectively. The natural frequencies of the three measurement units are 11.4 kHz, 11.4 kHz, and 13.2 kHz. It is indicated that excellent performance is achieved by the position-independent pure axial deformation piezoresistive beam.

## References

[1] Huang C-L, Yang S-C (2020). Sensorless vibration harmonic estimation of servo system based on the disturbance torque observer. IEEE Trans. Ind. Electron..

[2] Kim H, Kerrigan S, Bourham M, Jiang X (2021). AlN single crystal accelerometer for nuclear power plants. IEEE Trans. Ind. Electron..

[3] Liu Z, Song J (2018). A low-cost calibration strategy for measurement-while-drilling system. IEEE Trans. Ind. Electron..

[4] Yang J, Chao L (2020). Motion characteristic recognition of transmission lines based on inertial measurement. IEEE Trans. Ind. Electron..

[5] Zhang, L., Lu, J., Takagi, H. & Maeda, R. Frontside-micromachined planar piezoresistive vibration sensor: evaluating performance in the low frequency test range. *Aip Adv.* **4**, 10.1063/1.4862253 (2014).

[6] Roy AL, Sarkar H, Dutta A, Bhattacharyya TK (2014). A high precision SOI MEMS-CMOS + /- 4 g piezoresistive accelerometer. Sens. Actuators A-Phys..

[7] Roy AL, Bhattacharyya TK (2015). Design, fabrication and characterization of high performance SOI MEMS piezoresistive accelerometers. Microsyst. Technol.-Micro- Nanosyst.-Inf. Storage Process. Syst..

[8] Wung T-S, Ning Y-T, Chang K-H, Tang S, Tsai Y-X (2015). Vertical-plate-type microaccelerometer with high linearity and low cross-axis sensitivity. Sens. Actuators A-Phys..

[9] Wang, P. et al. A piezoresistive micro-accelerometer with high frequency response and low transverse effect. *Measurement Sci. Technol.* **28**, 10.1088/1361-6501/28/1/015103 (2017).

[10] Han J, Zhao Z, Niu W, Huang R, Dong L (2018). A low cross-axis sensitivity piezoresistive accelerometer fabricated by masked-maskless wet etching. Sens. Actuators A-Phys..

[11] Biswas S, Gogoi AK (2019). A wearable piezoresistive microaccelerometer with low cross-axis sensitivity for neurological disease diagnosis. Aeu-Int. J. Electron. Commun..

[12] Roylance LM, Angell JB (1979). A batch-fabricated silicon accelerometer. IEEE Trans. Electron Devices.

[13] Suminto, J. T. & Ieee. in *IEEE 9th Annual International Workshop on Micro Electro Mechanical Systems—An Investigation of Micro Structures*, *Sensors, Actuators, Machines and Systems*. 180–185 (IEEE, 1996).

[14] Lim MK, Du H, Su C, Jin WL (1999). A micromachined piezoresistive accelerometer with high sensitivity: design and modelling. Microelectron. Eng..

[15] Huang SS (2005). A high-performance micromachined piezoresistive accelerometer with axially stressed tiny beams. J. Micromech. Microeng..

[16] Yuan Y, Du H, Wang S (2010). A miniature in-plane piezoresistive MEMS accelerometer for detection of slider off-track motion in hard disk drives. Microsyst. Technol.-Micro- Nanosyst.-Inf. Storage Process. Syst..

[17] Xu Y (2016). Analysis and design of a novel piezoresistive accelerometer with axially stressed self-supporting sensing beams. Sens. Actuators A-Phys..

[18] Xu, Y. et al. A novel piezoresistive accelerometer with SPBs to improve the tradeoff between the sensitivity and the resonant frequency. *Sensors* **16**, 10.3390/s16020210 (2016). 10.3390/s16020210 PMC4801586 26861343

[19] Dong P (2008). High-performance monolithic triaxial piezoresistive shock accelerometers. Sens. Actuators A-Phys..

[20] Hsieh, H.-S., Chang, H.-C., Hu, C.-F., Cheng, C.-L. & Fang, W. A novel stress isolation guard-ring design for the improvement of a three-axis piezoresistive accelerometer. *J. Micromechan. Microeng.* **21**, 10.1088/0960-1317/21/10/105006 (2011).

[21] Song, J., He, C., Wang, R., Xue, C. & Zhang, W. A mathematical model of a piezo-resistive eight-beam three-axis accelerometer with simulation and experimental validation. *Sensors* **18**, 10.3390/s18113641 (2018). 10.3390/s18113641 PMC6263867 30373206

[22] Wang, Y., Zhao, X. & Wen, D. Fabrication and characteristics of a three-axis accelerometer with double L-shaped beams. *Sensors* **20**, 10.3390/s20061780 (2020). 10.3390/s20061780 PMC7146347 32213816

[23] Alunda, B. O. & Lee, Y. J. Review: cantilever-based sensors for high speed atomic force microscopy. *Sensors* **20**, 10.3390/s20174784 (2020). 10.3390/s20174784 PMC7506678 32854193

[24] Liu Y, Zhao Y, Wang W, Sun L, Jiang Z (2013). A high-performance multi-beam microaccelerometer for vibration monitoring in intelligent manufacturing equipment. Sens. Actuators.-Phys..

[25] Narasimhan, V., Li, H. & Jianmin, M. Micromachined high-g accelerometers: a review. *J. Micromechan. Microeng.* **25**, 10.1088/0960-1317/25/3/033001 (2015).

[26] Sankar, A. R., Lahiri, S. K. & Das, S. Performance enhancement of a silicon MEMS piezoresistive single axis accelerometer with electroplated gold on a proof mass. *J. Micromechan. Microeng.* **19**, 10.1088/0960-1317/19/2/025008 (2009).

